# Human Cytomegalovirus-Induced Degradation of CYTIP Modulates Dendritic Cell Adhesion and Migration

**DOI:** 10.3389/fimmu.2017.00461

**Published:** 2017-04-21

**Authors:** Linda Grosche, Christina Draßner, Petra Mühl-Zürbes, Lisa Kamm, Vu Thuy Khanh Le-Trilling, Mirko Trilling, Alexander Steinkasserer, Christiane S. Heilingloh

**Affiliations:** ^1^Department of Immunomodulation, University Hospital Erlangen, Erlangen, Germany; ^2^Institute for Virology, University Hospital Essen, Essen, Germany

**Keywords:** dendritic cells, HCMV, CYTIP, proteasomal degradation, adhesion, migration

## Abstract

As potent antigen-presenting cells, dendritic cells (DCs) are essential for the initiation of effective antiviral immune responses. Viruses and especially herpesviruses, which are able to establish lifelong persistence, exploit several immune evasion mechanisms targeting DC biology. Our group has previously shown that the α-herpesvirus herpes simplex virus type 1 inhibits mature DC (mDC) migration by inducing adhesion *via* degrading the cellular protein CYTIP (cytohesin-1 interacting protein), an important negative regulator of β2-integrin activity. In the present study, we extended our analysis to the β-herpesvirus human cytomegalovirus (HCMV), to investigate whether other *herpesviridae* also induce such modulations. Indeed, HCMV impairs mDC transwell migration capability following a CCL19-chemokine gradient, despite equivalent expression levels of the cognate chemokine receptor CCR7 at the corresponding time points post-infection. Remarkably, HCMV infection potently induced β2-integrin activity on mDCs. Furthermore, directly HCMV-infected mDCs, exhibiting viral gene expression, strongly adhere to fibronectin and ICAM-1, in contrast to mDCs lacking infection or viral gene expression. Interestingly, HCMV-positive mDCs display a proteasome-dependent degradation of CYTIP. Contrasting the migration toward CCL19, elevated expression levels of the chemokine receptor CXCR4 in HCMV-infected mDCs were associated with functional CXCL12-chemotaxis under the herein used conditions. In summary, our results show that HCMV shapes mDC adhesion to compromise migration toward CCL19, but retaining CXCL12 responsiveness. Thus, we hypothesize that a preferred migration pattern toward the bone marrow, but not to secondary lymphoid organs, could ultimately cause a failure in the induction of potent antiviral immune responses.

## Introduction

Dendritic cells (DCs) are very potent antigen-presenting cells operating at the interface of innate and adaptive immunity specialized for activation of lymphocytes, especially naïve T-cells (1–3). Importantly, DCs reside immobilized in an immature state in peripheral tissues (4) until maturation is induced by stimulation *via* pattern recognition receptors, antigen uptake, or specific pro-inflammatory cytokines (5). These maturing DCs upregulate the expression of MHC I and II as well as of costimulatory molecules (6). Furthermore, the chemokine receptors CCR7 and CXCR4 are highly expressed on mature DCs (mDCs) (7–10). CXCR4 binds the widely expressed chemokine CXCL12 (SDF-1), homing the cell, e.g., to the bone marrow where the highest expression is found (11, 12). In contrast, CCR7 responds to the chemokines CCL19 and CCL21, highly expressed in secondary lymphoid organs (SLOs), directing mDCs to SLOs for T cell activation (13, 14).

Leukocytes and especially DCs are very motile cells circulating through different tissues and lymphoid organs. In general, migration requires multiple changes in cytoskeleton architecture and cell–substrate interactions (15), regulated, e.g., by chemokine perception and signaling with rearrangement of the cytoskeleton and modulation of adhesion (16–18). Integrins are heterodimeric transmembrane receptors that mediate adhesion and comprise a very high diversity by the combination of α- and β-chains resulting in 24 members in mammalia, all possessing different ligands and binding affinities (19). Leukocytes express integrins of the β1-, β2-, β3- and β7-families, while β2- and β7-integrins are restricted to these cells (20). The β2-integrins consist of the β-subunit cluster of differentiation (CD) 18 that associates with one of the four different α-chains to form LFA-1 (CD11a/CD18 or αLβ2), Mac-1 or CR3 (CD11b/CD18 or αMβ2), p150.95 (CD11c/CD18 or αXβ2) and αDβ2 (CD11d/CD18) (21, 22). Expression of the latter ones is restricted to specific leukocyte subsets, while LFA-1 is constitutively expressed on all leukocytes, thus playing essential roles in controlling adhesion and cellular interactions (21, 23–25). Like all other integrins, LFA-1 dynamically switches between its active and inactive conformation, mediated by outside-in and inside-out signaling (19, 26). Only two proteins talin and cytohesin-1 are currently known to modulate LFA-1 activity by direct binding to its cytoplasmic CD18 tail (27). Interestingly, the latter one was reported to be specific for β2-integrins and to be predominantly expressed in hematopoietic cells (28, 29). Cytohesin-1 interacting protein (CYTIP), a direct interactor of cytohesin-1, is expressed by hematopoietic cells and upregulated during DC maturation (30–32). One important function of CYTIP is to abrogate cytohesin-1-induced activation of LFA-1. Cytohesin-1 directly interacts with membrane associated phosphatidylinositol-3,4,5-trisphosphate (PIP3), produced by phosphoinositide 3-kinase (33), and the intracellular CD18 domain of LFA-1 resulting in increased LFA-1 affinity, promoting adhesion to its ligands (28, 29, 34). CYTIP reverses these interactions by binding to cytohesin-1, with subsequent translocation of the cytohesin-1/CYTIP-complex to the cytosol, thereby diminishing LFA-1 affinity and ultimately adhesion (30).

The β-herpesvirus human cytomegalovirus (HCMV) exhibits seroprevalences of 45% up to almost 100% depending on age, gender and socioeconomic situation (35). While the primary infection of healthy adult individuals is usually subclinical, infections of immune-immature fetus or neonates as well as immunocompromised patients frequently leads to severe symptoms with high morbidity and mortality (36). Permissive target cells for HCMV replication are fibroblasts, smooth muscle cells, epithelial cells, and endothelial cells (37, 38). Apart from the productive lytic replication, herpesviruses establish lifelong latency in the host, despite the presence of an immune response. Regarding HCMV, CD34^+^ hematopoietic progenitor cells (39–41) and CD14^+^ monocytes (42, 43) are widely accepted reservoirs for latency.

Since DCs are important stimulators of antiviral immune responses, herpesviruses evolved several strategies to interfere with DC functions. One example is the herpes simplex virus type 1 (HSV-1)-induced inhibition of mDC migration. This is mediated *via* the downregulation of CCR7 and CXCR4 surface expression as well as the activation of LFA-1 *via* the degradation of CYTIP leading to significantly induced adhesion of mDCs (44, 45). However, it was unknown whether this represents a unique immune evasion mechanism of HSV-1 or if other members of the *herpesviridae* family including HCMV also regulate mDC adhesion and migration, which would imply an analogous or even evolutionary conserved immune evasion mechanism.

In the present study, we provide evidence that HCMV modulates mDC adhesion and migration. We show a differential regulation of CCR7 and CXCR4 upon HCMV infection of mDCs. Moreover, HCMV induces activation of β2-integrins *via* proteasomal degradation of CYTIP in mDCs in which HCMV established viral gene expression. This leads to increased adhesion of infected mDCs to ICAM-1 and fibronectin, which in turn influences the mDC migration capability. Hence, we suggest that this immune evasion mechanism has been evolved by at least one prototypic member of the α- as well as β-*herpesviridae* family, to hamper the induction of potent antiviral immune responses.

## Materials and Methods

### Generation of DCs

Mature DCs were generated from peripheral blood mononuclear cells (PBMCs) from healthy donors as reported earlier (46). PBMCs were isolated by sedimentation with Lymphoprep (Nycomed Pharma) as previously described (47). Cells were cultured in DC medium (RPMI 1640 medium [BioWhittaker] supplemented with 100 U penicillin ml^−1^, 100 mg streptomycin ml^−1^ [Sigma], 1% (v/v) autologous serum, 2 mM l-glutamine [Cambrex BioScience] and 10 mM HEPES pH 7.5 [Sigma-Aldrich]) and were allowed to adhere on standard tissue flasks (Nunc) for 1 h. Subsequently, non-adherent cells were rinsed off with RPMI, and differentiation to DCs was performed as described elsewhere (48). Briefly, iDCs were generated by addition of 800 U of granulocyte-macrophage colony-stimulating factor (GM-CSF) ml^−1^ (Miltenyi) and 250 U IL-4 ml^−1^ (Miltenyi) in DC medium. Maturation of DCs was induced by adding 40 U GM-CSF ml^−1^, 250 U IL-4 ml^−1^, 10 ng TNF-α ml^−1^ (Peprotech), 1 mg prostaglandin E2 ml^−1^ (Pfizer), 200 U IL-1β ml^−1^ (Cell-Genix) and 1,000 U IL-6 ml^−1^ (Cell-Genix) to the medium. After 2 days, DC maturation status was analyzed by flow cytometry.

### Cells and Viruses

The HSV-1/17+/CMV-EGFP/UL43 strain, used in this study, contains the EGFP reporter cassette under control of the CMV promoter inserted into the *UL43* locus of HSV-1 strain 17+. Virus stock preparation and titer determination were performed as described previously (49). The HSV-1 strain was obtained from BioVex.

For HCMV infection, the HCMV TB40E/IE2-EYFP strain (50) expressing IE2-EYFP fusion protein (kindly provided by Thomas Stamminger, Institute for Clinical and Molecular Virology, Erlangen) or HCMV-TB40E-green fluorescence protein (GFP) was used. HCMV stocks were prepared on ARPE-19 cells, followed by propagation on human fibroblasts (HFF or MRC-5 cells), cultured in Dulbecco’s Modified Eagel’s Medium (DMEM; Lonza) supplemented with 10% (v/v) FCS (PAA), 2 mM l-glutamine (Lonza), 100 U penicillin ml^−1^ (Lonza) and 100 mg streptomycin ml^−1^ (Lonza). Virus-containing supernatants were harvested when all cells showed cytopathic effects. Remaining adherent cells were scraped from the flask and added to the supernatant. Cellular debris as well as intact cells were collected by centrifugation and homogenized using a tissue grinder (VWR) to release intracellular virions. Cellular debris in the virus suspensions were finally removed by centrifugation, and virus-containing supernatant was stored at −80°C. Titration of the respective HCMV strains was carried out as described elsewhere (51). Ultraviolet (UV)-inactivated virus (HCMV-UV) was generated by irradiation of HCMV-containing suspensions with defined doses of UV light (1,200 J/cm^2^).

### HSV-1 Infection of mDCs

Mature DCs were harvested and washed in PBS. Cells (1 × 10^6^) were resuspended in 300 µl RPMI 1640 (Lonza) supplemented with 20 mM HEPES (*N*-2-hydroxyethylpiperazine-*N*-2-ethanesulfonic acid, Lonza). Cells were then incubated with HSV-1 at a multiplicity of infection (MOI) of 2 for 1 h at 37°C and shaking at 300 rpm. After collecting the cells by centrifugation, they were cultured in DC medium supplemented with 40 U GM-CSF ml^−1^ and 250 U IL-4 ml^−1^ until further use. For proteasomal inhibition, 10 µM of MG-132 (Enzo Life Science) or dimethyl sulfoxide (Sigma-Aldrich) as control was added 1 hour post-infection (hpi).

### HCMV Infection of mDCs

Mature DCs were collected and washed once in PBS. Cells (5 × 10^5^) were resuspended in the respective volume of HCMV virus stock at an MOI of 3. For mock condition, mDCs were resuspended in the respective volume of DMEM supplemented with 10% FCS, 2 mM l-glutamine, 100 U penicillin ml^−1^ and 100 mg streptomycin ml^−1^. For infection, mDCs were centrifuged at 1,500 × *g* at room temperature for 1 h. Afterward, cells were cultured in DC medium containing 40 U GM-CSF ml^−1^ and 250 U IL-4 ml^−1^ in a final volume of 1 ml.

### Mg/EGTA Treatment of mDCs

For induction of integrin activity, mDCs were treated as described previously (52). Briefly, cells were resuspended in RPMI 1640 supplemented with 20 mM HEPES, 5 mM MgCl_2_ and 1 mM ethylene glycol-bis(β-aminoethyl ether)-*N*,*N*,*N*′,*N*′-tetraacetic acid (EGTA). After 30-min incubation at 37°C, cells were subjected to flow cytometric analyses, fibronectin adhesion, or transwell migration assay.

### Flow Cytometric Analyses, Fluorescence-Activated Cell Sorting (FACS) and Antibodies Used

To verify purity and maturation status of DCs, flow cytometric analyses were performed. For HCMV infection experiments, surface expression of different surface proteins was analyzed by staining with the following labeled monoclonal antibodies: CCR7-PE/Cy7 (clone G043H7, BioLegend), CXCR4-APC (clone 12G5, BioLegend), CD11a-PE (clone TS2/4, BioLegend), CD11b-APC-Cy7 (clone ICRF44, BD Biosciences), CD11c-PeCy5 (clone B-ly6, BD Biosciences), CD18-APC (clone CBR LFA-1/2, BioLegend), anti-activated CD11/CD18 clone mAB24 (Hycult Biotech), or the isotype control mouse IgG1 κ (clone P3.6.2.8.1, eBiosciences) with subsequent incubation with an AlexaFluor647-labeled secondary antibody (Invitrogen). Indicated antibodies were resuspended in PBS supplemented with 2% (v/v) FCS. Intracellular flow cytometry analyses for detection of CYTIP and cytohesin-1 (using the mAbs rat anti-CYTIP clone 2F9 and rat anti-cytohesin-1 clone 7H2, respectively; kind gift from E. Kremmer, Helmholtz Center Munich, Germany) were performed using Cytofix/Cytoperm kit (BD Biosciences) according to the manufacturer’s instructions, with subsequent incubation with a PE-labeled secondary antibody (clone Poly4054, BD Biosciences). For life/dead discrimination, the cells were additionally stained using the LIVE/DEAD Fixable Violet dead cell stain kit (Life Technologies). Staining procedures were performed at 4°C and prior to flow cytometry analyses cells were fixed in PBS supplemented with 2% (v/v) FCS and 2% (w/v) PFA (Alfa Aeasar). Gating on HCMV-positive or -negative mDCs was based on their GFP/YFP signal. Flow cytometric measurements were performed using a BD FACS Canto II (BD Biosciences), and data were analyzed using FCS express 5. FACS for HCMV-positive or -negative mDCs was performed using a BD Aria FACS cell sorter (BD Biosciences).

### Western Blot Analyses and Antibodies Used

Cell lysates were prepared with NP-40 lysis buffer (10% [v/v] glycerol; 2 mM EDTA, pH 8; 137 mM NaCl; 50 mM Tris pH 7.2; 0.5% [v/v] NP-40) supplemented with 20 mM sodium fluoride, 2 mM phenylmethanesulfonyl fluoride, and 2 mM sodium orthovanadate. After incubation for 20 min on ice, lysates were cleared by centrifugation at 15,900 × *g* for 20 min at 4°C. Subsequently, 4× Laemmli buffer (125 mM Tris-HCl pH 6.8, 4% SDS, 20% [v/v] glycerin, bromophenol blue, 10% [v/v] 2-mercaptoethanol) was added, and samples were boiled at 95°C for 5 min. After separating the proteins by SDS-PAGE, Western blotting on a nitrocellulose membrane (GE Healthcare) was performed. Membranes were blocked with 1× RotiBlock (CarlRoth) and incubated with the indicated primary antibodies in 1× RotiBlock at 4°C overnight. The following primary antibodies were used: CYTIP and cytohesin-1 (mAbs rat anti-CYTIP clone 2F9 and rat anti-cytohesin-1 clone 7H2, respectively), anti-IE2 (clone 12E2, Santa Cruz Biotechnology), anti-ICP0 (clone 11060, Santa Cruz Biotechnology), and anti-GAPDH (clone 6C5, Millipore). After incubation with the appropriate secondary HRP-labeled antibody (Cell Signaling Technology) for 1 h at room temperature, signals were detected using Amersham ECL Prime Western Blotting Detection Reagent (GE Healthcare).

### Fibronectin Adhesion Assay

Fibronectin coating of 96-well Maxisorp plates (Nunc) was performed using 20 µg fibronectin ml^−1^ (Sigma-Aldrich) in PBS with incubation at 4°C overnight. Subsequently, wells were blocked with 0.01% (w/v) gelatin (Sigma-Aldrich) in PBS for 2 h at room temperature. Directly before adhesion wells were washed in PBS. After harvesting 24 hpi, mDCs were washed once in PBS and adjusted to 4 × 10^5^ cells per ml in RPMI 1640. Per well, 100 µl of the DC suspension was added, and cells were allowed to adhere for 45 min at 37°C. Subsequently, supernatant was removed, and non-adherent mDCs were washed off with RPMI 1640. Adherent fraction was rinsed with PBS and fixed with 2% (w/v) PFA. To determine input conditions the respective volume of the cell suspension was preserved before initiation of the adhesion assay. Each experimental condition was performed with a total cell number of 2 × 10^5^ cells. Infection rates before and after adhesion were determined using flow cytometry. To calculate the number of adherent cells, their β-glucuronidase activity was measured as described previously (44). Briefly, cells were resuspended in 25 µl PBS and 5 µl 1% TritonX-100 in PBS was added. After 20-min incubation at room temperature, cell debris was removed by centrifugation at 18,400 × *g* for 1 min, and lysates were incubated for 6 h at 37°C with 75 µl 10 mM 4-nitrophenyl-β-d-glucuronide (Sigma-Aldrich) solved in 100 mM sodium acetate, pH 4. Subsequently, 100 µl 0.4 M glycine, pH 10, was added, and the absorbance at 405 nm was measured using a Victor^2^ multilabel counter (PerkinElmer, Jugesheim, Germany). The number of adherent cells was calculated *via* a separate standard curve for each sample using cells of the input condition.

### ICAM Adhesion Assay

For ICAM-1 coating of 96-well Maxisorp plates (Nunc), wells were coated with 4 µg goat anti-human IgG (Fc specific) per ml (Jackson ImmunoResearch Laboratories) in PBS at 4°C overnight. After blocking with 1% (w/v) BSA in PBS at 37°C for 30-min, wells were washed with PBS and subsequently incubated with 500 ng ICAM-1-Fc ml^−1^ in PBS for 1 h at 37°C. Adhesion of mDCs and quantification of infection rates were performed as described above for the fibronectin adhesion assay.

### Transwell Migration Assay

Membranes of transwell inserts with a pore size of 5 µm (Corning Costar) were coated with 20 µg fibronectin ml^−1^ at 4°C overnight. Transwell migration assays were performed as described elsewhere (44). In brief, mDCs were harvested 24 hpi, washed once in PBS and adjusted to 2 × 10^6^ cells per ml in DC medium. To eliminate cell clusters, cells were passed with a syringe three times through a 20 gage needle. Transwell inserts were equilibrated with 100 µl DC medium, while 600 µl of medium was added to the wells of a 24-well plate. For migration assay, 2 × 10^5^ cells were seeded into the inserts and allowed to migrate at 37°C for 2 h toward chemokine gradients of CCL19 or CXCL12 (100 ng ml^−1^, Tebu-Bio GmbH), added to the lower compartment. Additionally, the respective amount of cells was preserved as starting condition. Afterward, migrated DCs were harvested from the lower chamber. Cell numbers and infection rates before and after migration were determined using flow cytometry.

### Immunofluorescence Confocal Microscopy

Mock- or HCMV-infected mDCs were allowed to adhere on poly-l-lysin (Sigma-Aldrich)-coated glass cover slips. Cells were fixed using 4% paraformaldehyde and subsequently permeabilized using 0.2% Triton-X-100. Blocking was performed with 1% BSA in PBS. Antibodies used for immunofluorescence staining were mAbs rat anti-CYTIP clone 2F9 and rat anti-cytohesin-1 clone 7H2 and anti-rat AlexaFluor555-conjugated secondary antibody (Invitrogen, Carlsbad, CA, USA). For mounting and additional nuclear staining, Roti^®^-Mount FluorCare DAPI (Carl Roth) was used. Confocal microscopy was performed using a LSM780 microscope (Zeiss, Oberkochen, Germany).

### Statistical Analysis

For determination of significance, data sets of two conditions were analyzed using two-tailed Student’s *t*-test. For multiple data sets, one-way analysis of variance was applied. Results are displayed as mean ± SD. Significance was accepted for *p*-values less than 0.05. **** indicates *p* ≤ 0.0001; ***p ≤ 0.001; **p ≤ 0.01; *p ≤ 0.05; and ns, not significant.

## Results

### HCMV Reduces mDC Transwell Migration toward CCL19, but Not CXCL12

As migration of DCs to SLOs is pivotal for induction of immune responses by activation of naïve T cells, DCs constitute potential targets for viral immune evasion strategies. Previously, we showed that HSV-1 efficiently infects monocyte-derived mDCs and inhibits transwell migration toward the SLO-expressed chemokines CCL19 and CXCL12 (44). In order to investigate whether this modulation of migration is a unique mechanism of HSV-1 or also exist in other *herpesviridae*, we analyzed the influence of HCMV, which represents the prototype of the β-herpesvirus family.

**Figure 1:**
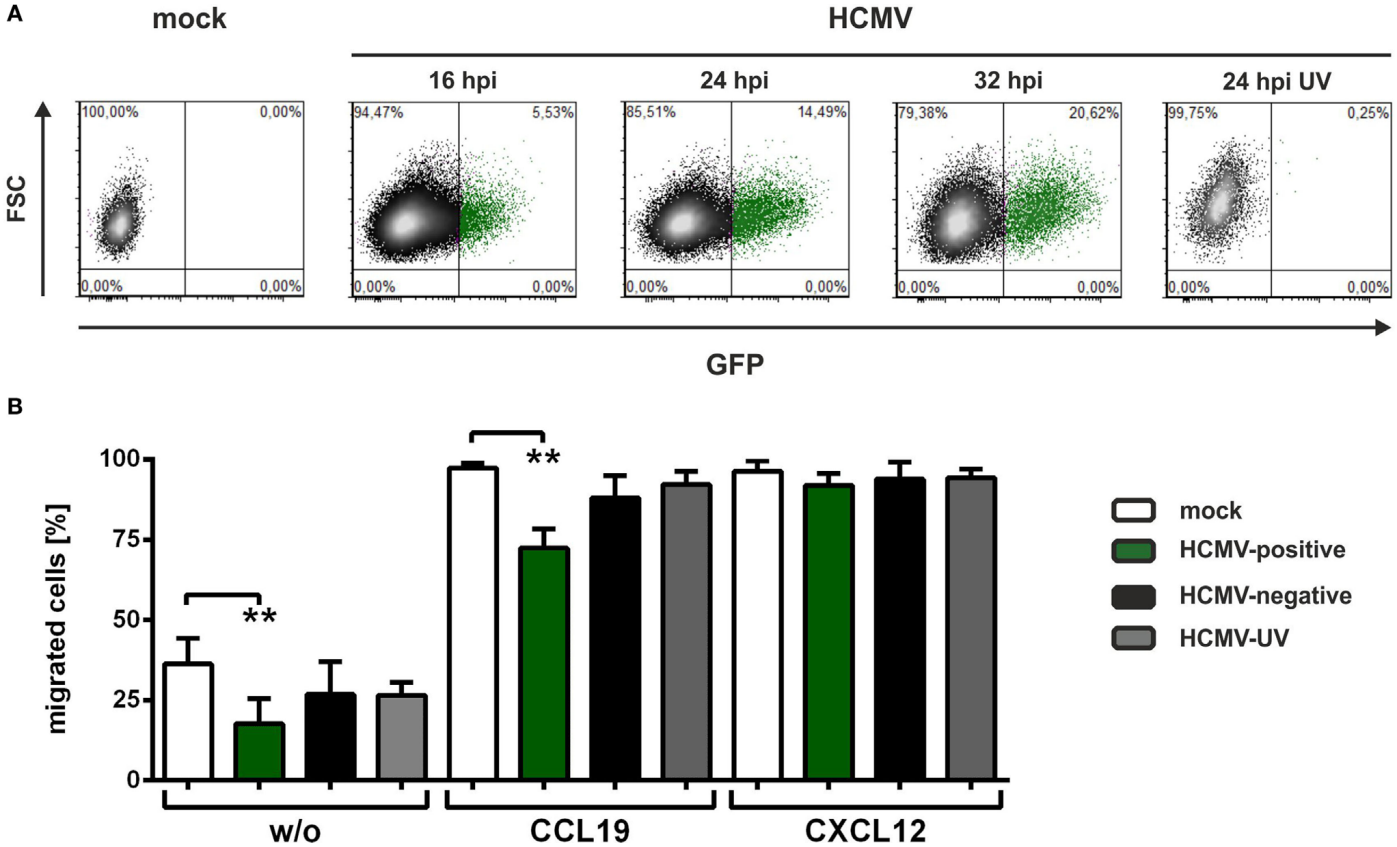
**Human cytomegalovirus (HCMV)-infected mature DCs (mDCs) show decreased transwell migration toward CCL19**. **(A)** Mature dendritic cells were mock-, HCMV- or HCMV-ultraviolet (UV)-infected and analyzed for infection rates at the indicated time points. Mock or HCMV/green fluorescence protein (GFP)-negative mDCs are indicated as black dots, while HCMV/GFP-positive cells are marked in green. **(B)**. Mock- (white columns), HCMV-infected mDCs (HCMV-positive cells are depicted in green and HCMV-negative cells in black columns), and HCMV-UV-infected mDCs (gray columns) were subjected to a transwell migration assay on fibronectin-coated transwell inserts 24 hpi. To induce chemotaxis the chemokines CCL19 or CXCL12 (100 ng/ml) were added to the lower wells. As control no chemokine was added for spontaneous migration, indicated as “w/o.” Migration was evaluated after 2 h, and the percentage of migrated mDCs was calculated in the lower well by determining cell number and infection rates of migrated cells in relation to the respective starting condition. The experiment was performed at least three times with cells from different healthy donors. Significant changes (** = *p* < 0.01) are indicated by asterisks.

For all infection experiments performed in the present study, the endotheliotropic strain HCMV-TB40E expressing GFP or an IE2-EYFP (enhanced yellow fluorescent protein) fusion protein was used. The use of the GFP/YFP-tagged HCMV-TB40E strain allows the discrimination of HCMV-positive mDCs that express viral-encoded genes and HCMV-negative mDCs, which were exposed to viral particles and antigens but did not express HCMV-encoded genes. Figure 1A shows a time course analysis of HCMV-infected monocyte-derived mDCs. At the indicated time points, cells were analyzed *via* flow cytometry for GFP/YFP fluorescence, and approximately 15% of mDCs expressed EGFP as undeniable indicator of HCMV-induced gene expression 24 hpi, with increasing percentages at later time points (Figure 1A). Furthermore, mDCs were infected with UV-inactivated HCMV (HCMV-UV) and analyzed 24 hpi to ensure UV-irradiation efficiency.

To assess the influence of HCMV on mDC chemotaxis, we subjected mock-, HCMV- or HCMV-UV-infected mDCs 24 hpi to transwell migration assays using fibronectin-coated polycarbonate membranes (Figure 1B). After 2 h of migration without chemokine as well as toward CCL19 or CXCL12, cells in the lower well were counted and infection efficiency was determined before and after migration. No change in CXCL12-mediated chemotaxis was observed comparing mock- (white bar), HCMV-positive (green bar), HCMV-negative (black bar), and HCMV-UV-infected (gray bar) mDCs, indicating that HCMV does not compromise the general ability of mDCs to migrate to chemotactic gradients. However, spontaneous migration without chemokines (w/o) was significantly inhibited for HCMV-positive mDCs compared to mock condition. Interestingly, we observed a significantly reduced migration capability for HCMV-positive mDCs in CCL19-induced chemotaxis compared to mock cells, HCMV-negative as well as HCMV-UV-infected mDCs (Figure 1B).

### Surface Expression of CCR7 and CXCR4 in HCMV-Infected mDCs Does Not Explain Impaired Migration

**Figure 2:**
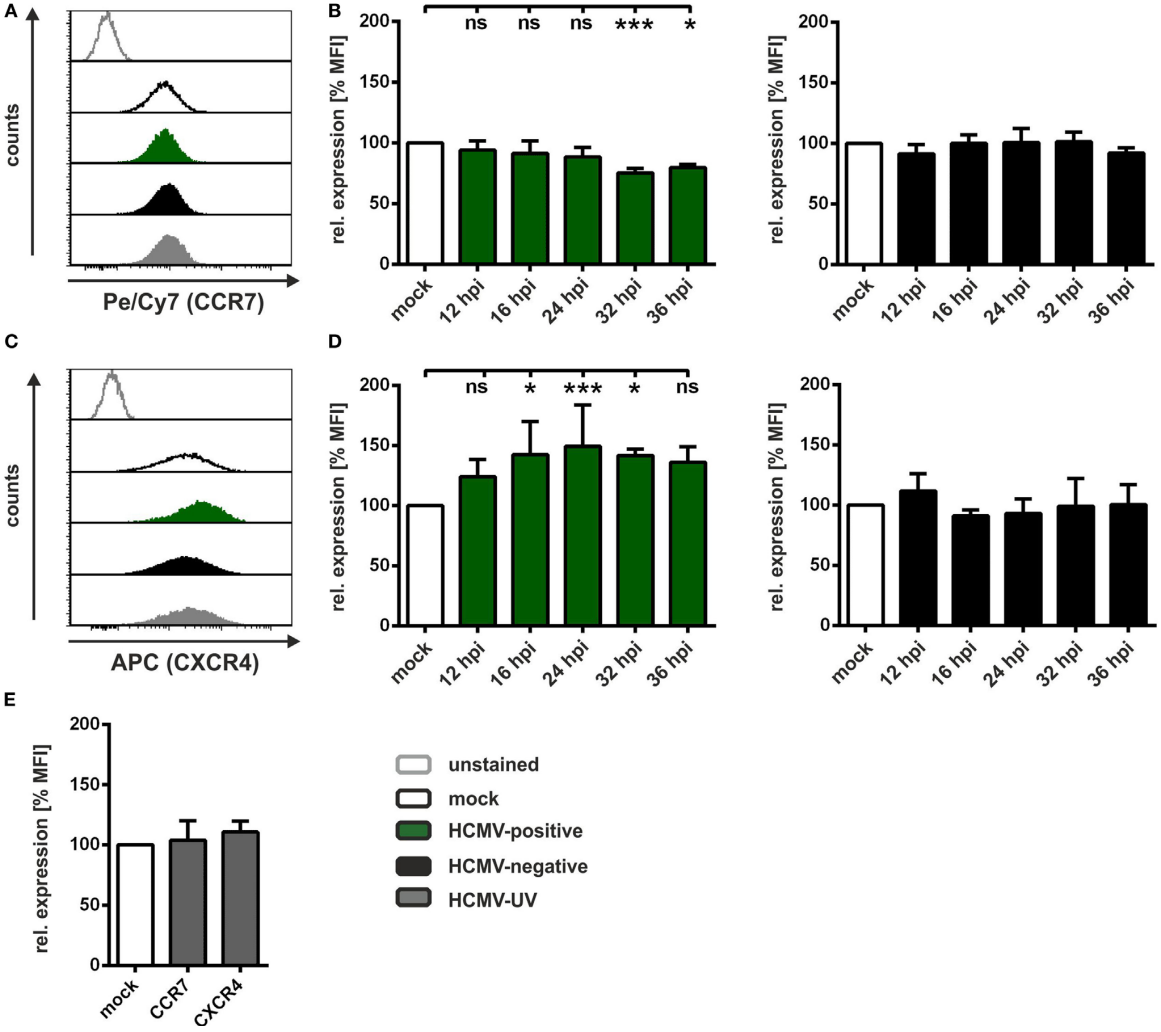
**Human cytomegalovirus (HCMV) differentially modulates CCR7 and CXCR4 surface expression on infected mature DCs (mDCs)**. Mock-, HCMV- or HCMV-ultraviolet (UV)-infected mDCs were harvested at the indicated time points and stained with antibodies specific for CCR7 and CXCR4 for flow cytometric analyses. **(A)** Representative data of one donor are shown for surface expression of CCR7 and **(C)** CXCR4 24 hpi. Mock cells (black histogram), HCMV-positive cells (filled green histogram), HCMV-negative cells (filled black histogram), and HCMV-UV-infected cells (filled gray histogram) are depicted. **(B)** Time kinetics of CCR7 and **(D)** CXCR4 surface expression are shown as mean fluorescence intensity (MFI) of HCMV-positive (green bars, left panel) and HCMV-negative mDCs (black bars, right panel) relative to mock cells (white bars, set to 100%). **(E)** CCR7 and CXCR4 surface expression of HCMV-UV-infected mDCs (gray bars) 24 hpi are shown as MFI relative to mock condition (white bar, set to 100%). The experiment was performed at least three times with cells from different healthy donors. Significant changes (*** = *p* < 0.001; * = *p* < 0.05) are indicated by asterisks, non-significant changes (*p* > 0.05) as “ns.”

Chemotaxis in a transwell system relies on the expression of the respective receptors on the cell surface. Our group previously reported that HSV-1 induces significant downregulation of surface expression of the receptors CCR7 and CXCR4, which are required to recognize CCL19 and CXCL12, respectively (44). To determine alterations in surface expression of both chemokine receptors on HCMV-infected mDCs, we performed time course experiments, with subsequent flow cytometric analyses (Figure 2). The results of one representative experiment of mock, HCMV-positive, HCMV-negative, and HCMV-UV-infected mDCs 24 hpi are shown in Figures 2A,C, while the results of at least three independent experiments in a time kinetic are summarized in Figures 2B,D. We demonstrate that CCR7 surface expression is unaffected until 24 hpi—the time frame in which the transwell assay was conducted—followed by a weak downregulation at later time points only in HCMV-positive mDCs (Figure 2B, left panel). Remarkably, in contrast to the downregulation of CCR7, HCMV even induced CXCR4 expression already 12 hpi, peaking at 24 hpi with an induction of approximately 150% only observed in HCMV-positive mDCs (Figure 2D, left panel). No changes in CCR7 and CXCR4 surface expression were detected on HCMV-negative (Figures 2B,D, right panels) as well as on HCMV-UV-infected mDCs (Figure 2E).

The surface disposition of these two chemokine receptors was altered on HCMV-positive mDCs but failed to explain their impaired transwell migration observed within the first 24 h of mDC infection. Hence, additional mechanism(s), apart from meaningful downregulation of CCR7 surface expression, were considered as cause for the observed inhibition of transwell migration toward CCL19.

### HCMV Induces mDC Adhesion by Increasing β2-Integrin Activity Leading to Impaired CCL19-Directed Transwell Migration

Having demonstrated that HCMV does not downregulate surface expression of CCR7 until 24 hpi, we intended to elucidate the mechanism by which migration in this 2D system is inhibited. Apart from chemokine receptor expression, mDC adhesion on the fibronectin-coated transwell polycarbonate-membrane could be modulated by HCMV to inhibit migration. While fibronectin represents a prototypic ligand for a variety of cell adhesion molecules, belonging to β1-, β2-, β3- and β7-integrin families (53, 54), ICAM-1 is a specific ligand for the β2-integrins LFA-1 and Mac-1 (55).

**Figure 3:**
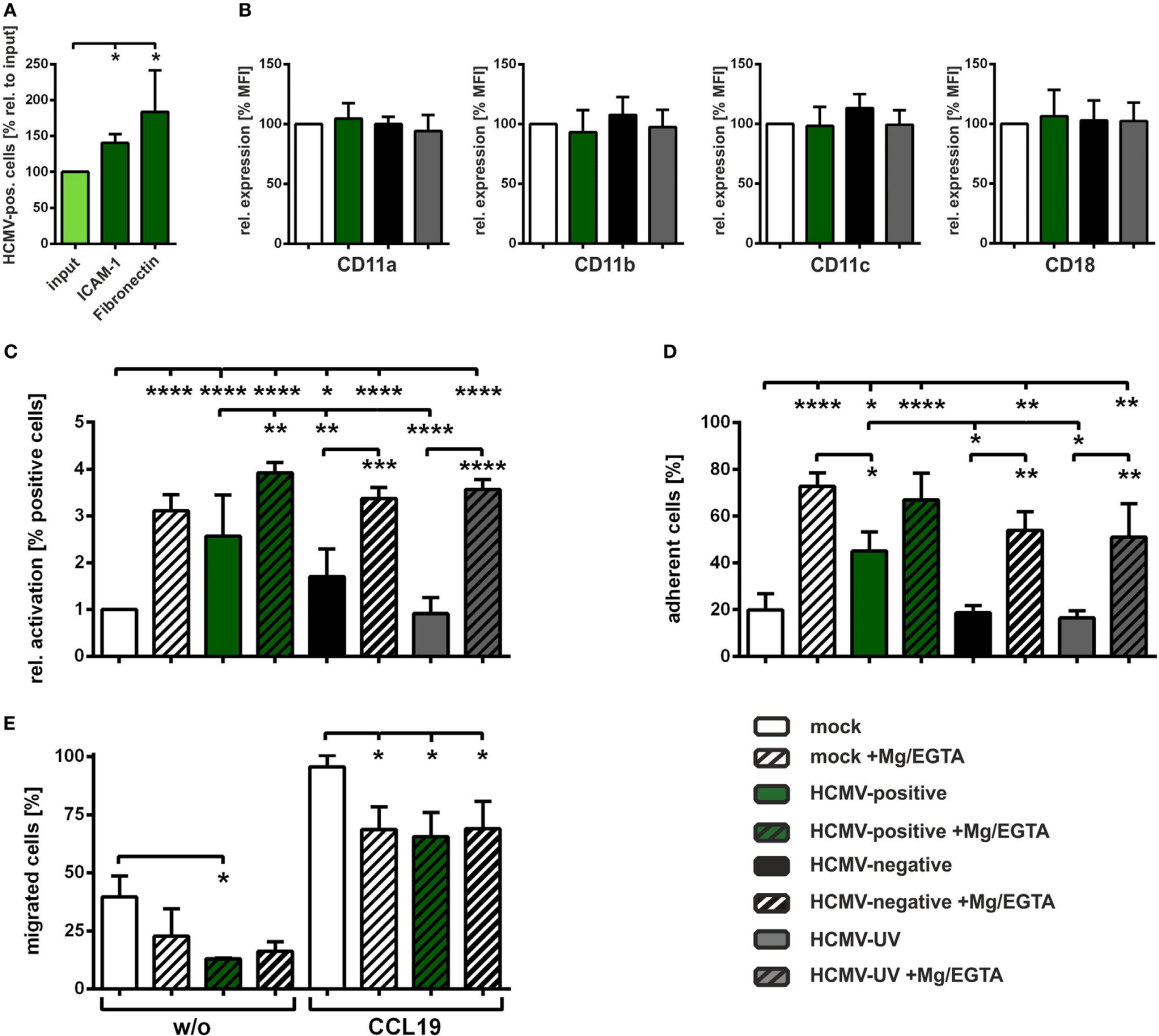
**Human cytomegalovirus (HCMV) increases activation of β2-integrins thereby inducing mature DCs (mDCs) adhesion and reducing mDC transwell migration**. **(A)** Mature dendritic cells (DCs) were HCMV-infected and subjected to ICAM-1 or fibronectin adhesion assays 24 hpi. Infection rates were determined before adhesion (input condition, light green bar) and after ICAM-1 (*n* = 5) or fibronectin (*n* = 3) adhesion (green bars). Infection rate before adhesion (input) was set to 100% and relative changes of infection rates in the adherent fractions are shown. **(B)** Mock-, HCMV-, or HCMV-ultraviolet (UV)-infected mDCs were harvested 24 hpi, stained with antibodies specific for CD11a/b/c or CD18 and analyzed *via* flow cytometry. Uninfected mock cells (white bar) were set to 100% and relative expression as mean fluorescence intensity for HCMV-positive (green bars), HCMV-negative (black bars), and HCMV-UV-infected (gray bars) mDCs are shown (*n* = 3). **(C)** Mock-, HCMV-, or HCMV-UV-infected mDCs were harvested 24 hpi and treated with (striped bars) or without Mg/EGTA (filled bars). Subsequently, cells were stained with an antibody specific for an epitope specifically present on activated CD11/CD18 (β2) integrins and subjected to flow cytometric analyses. Mock (white bar) was set to “1,” and relative activation is shown for the indicated conditions. The experiment was performed at least three times with cells of different healthy donors. **(D)** Mature DCs were infected and treated with (striped bars) or without Mg/EGTA (filled bars) as described in **(C)** and subjected to fibronectin adhesion assay. Percentages of adherent cells are depicted for each condition (*n* = 3). **(E)** Mock- or HCMV-infected mDCs (24 hpi) were treated with Mg/EGTA (striped bars) and subjected to transwell migration assays. For induction of chemotaxis, CCL19 (100 ng/ml) was added to the lower wells, while spontaneous migration without chemokine was analyzed as control (w/o). Migration was evaluated after 2 h. The percentages of migrated mDCs were calculated in the lower well by determining cell number and infection rates of migrated cells in relation to the respective starting condition. Untreated mock cells are depicted as control (white bars). The experiment was performed with cells of three different healthy donors. Only significant changes (**** = *p* < 0.0001; *** = *p* < 0.001; ** = *p* < 0.01; * = *p* < 0.05) are marked by asterisks.

Therefore, we next analyzed adhesion of HCMV-infected mDCs on ICAM-1 or fibronectin-coated wells 24 hpi (Figure 3A). Input condition (light green bar) represents the infection rate before adhesion and was set to 100%. ICAM-1 as well as fibronectin increased the ratio of HCMV-positive cells among the adherent fractions (second and third green bar), demonstrating that HCMV-positive mDCs adhere stronger to both ligands.

To investigate whether this increased adhesion might be due to an enhanced expression of the β2-integrin subunits, CD11a, CD11b, CD11c or CD18, flow cytometric analyses of mock-, HCMV-, or HCMV-UV-infected mDCs were performed 24 hpi (Figure 3B). Expression of the indicated subunits was comparable between mock (white bars), HCMV-positive (green bars), HCMV-negative (black bars), and HCMV-UV-infected (gray bars) mDCs.

Since we previously demonstrated that increased ICAM-1 and fibronectin adhesion of HSV-1-infected mDCs is based on the induced β2-integrin activity (45), we next examined the activation status of β2-integrins after HCMV infection using an antibody which recognizes a conformational epitope only present on activated β2-integrins (56–58). As a positive control we treated the cells with Mg/EGTA to induce β2-integrin activity *via* alteration of divalent cation concentration, since it is known that Mg^2+^ promotes transition from the inactive into the active conformational state (52, 59, 60). Flow cytometric analyses of untreated mock (white bar) and HCMV-positive (green bar) mDCs revealed a significant induction of β2-integrin activity of approximately 2.5-fold, while HCMV-negative (black bar) mDCs show a less pronounced induction (Figure 3C). No differences in β2-integrin activity were observed comparing mock and HCMV-UV-infected mDCs (gray bar). As expected, treatment with Mg/EGTA showed a strong increase in β2-integrin activity in mock, HCMV-negative, and HCMV-UV condition. Interestingly, Mg/EGTA treatment of HCMV-infected cells further increased the activation status of β2-integrins on HCMV-positive mDCs compared to their untreated counterparts.

In order to analyze whether the observed increase in β2-integrin activity shown in Figure 3C correlates with induced adhesion, a fibronectin adhesion assay was conducted (Figure 3D). In agreement with the activation status of β2-integrins on HCMV-positive mDCs, we observed a significant induction in fibronectin adhesion compared to mock cells, while this was not detected for HCMV-negative and HCMV-UV-infected mDCs. Moreover, Mg/EGTA-treated cells adhered stronger to fibronectin compared to their respective untreated counterparts.

In order to evaluate whether the induced adhesion of HCMV-positive mDCs accounts for the reduced CCL19 transwell migration (Figure 1B), we performed a transwell migration assay with Mg/EGTA-treated mock cells to induce adhesion in the absence of viral infection (Figure 3E). Treatment with Mg/EGTA strongly reduced spontaneous and CCL19-directed chemotaxis comparable to the reduction observed for HCMV-positive mDCs (Figure 1B), while additional HCMV infection did not further reduce CCL19 transwell migration.

Therefore, we conclude that HCMV activates β2-integrins on infected mDCs to facilitate increased adhesion to fibronectin as well as ICAM-1, which in turn leads to reduced CCL19-mediated transwell migration.

### HCMV Infection of mDCs Leads to Decreased Expression Levels of CYTIP, the Negative Regulator of β2-Integrin Activation

**Figure 4:**
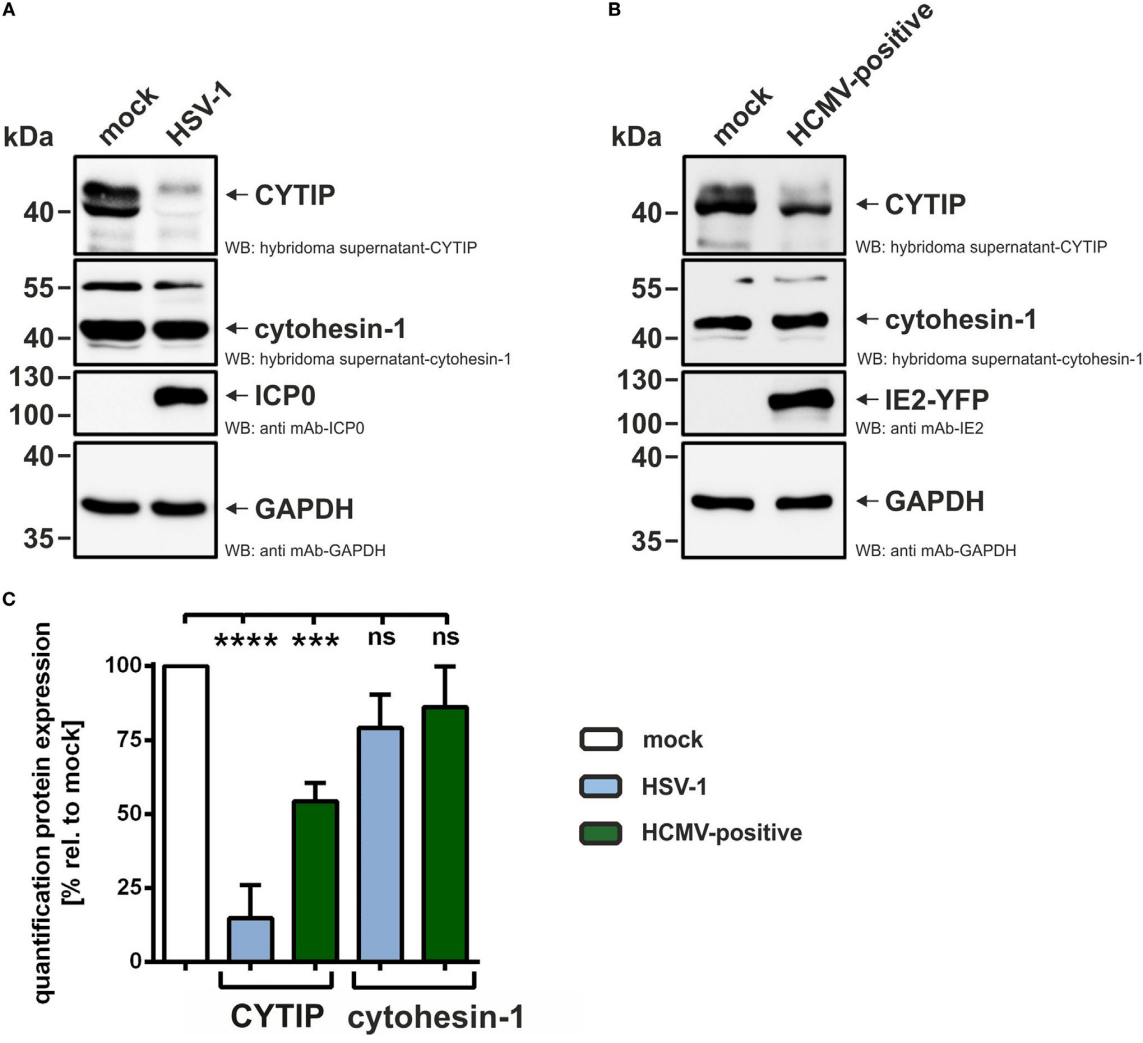
**Human cytomegalovirus (HCMV) infection of mature dendritic cells (mDCs) leads to decreased CYTIP expression levels**. **(A)** mDCs were either mock- or herpes simplex virus type 1 (HSV-1)-infected and harvested 24 hpi, **(B)** mock- or HCMV-infected and sorted based on their green fluorescence protein expression 24 hpi. **(A,B)** Protein lysates were prepared and subjected to Western blot analyses with antibodies specific for CYTIP, cytohesin-1, ICP0, IE2, or GAPDH. One representative experiment out of at least three is shown. **(C)** Quantification of CYTIP and cytohesin-1 protein expression levels in HSV-1-infected or HCMV-positive mDCs, summarizing at least three independent experiments. Expression of CYTIP and cytohesin-1 was normalized to the GAPDH control. Mock was set to 100%. Significant changes (**** = *p* < 0.0001; *** = *p* < 0.001) are marked by asterisks, non-significant changes (*p* > 0.05) as “ns.”

Next, we aimed to unravel the molecular mechanism of the observed β2-integrin activation on HCMV-positive mDCs. Cytohesin-1 is known to bind to the intracellular CD18 tail of β2-integrins and thereby causes a conformational change leading to their activation (28, 29, 34). Furthermore, it was demonstrated that the cellular protein CYTIP negatively regulates LFA-1 activity and consequently ICAM-1 adhesion (30, 45). In particular, using siRNA-mediated CYTIP ablation, it was previously shown that the loss of CYTIP suffices to induce mDC adhesion and reduce migration capability (45). Therefore, we next investigated cytohesin-1 and CYTIP expression in HCMV-infected mDCs. In this regard, mDCs were mock- or HCMV-infected and subjected to FACS, based on their GFP/YFP expression. As a control, HSV-1-infected mDCs were analyzed; however, due to the high infection efficiency of nearly 100%, cell sorting was dispensable in this case. Confirming previous results, CYTIP protein levels were lost in HSV-1-infected mDCs 24 hpi (Figure 4A shows one representative experiment and Figure 4C summarizes the quantification of at least three independent experiments). In contrast, protein expression of cytohesin-1, which is the positive regulator of LFA-1 activity, is only affected to a minor extent. Interestingly, also in HCMV-positive mDCs CYTIP protein expression levels are reduced to approximately 50% (24 hpi), while cytohesin-1 protein expression is not significantly influenced (Figures 4B,C).

Thus, these results provide evidence that HCMV induces downregulation of CYTIP in order to increase LFA-1 activation on the surface of infected mDCs leading to induced adhesion and reduced migration.

### HCMV Induces CYTIP Downmodulation Early after Infection

**Figure 5:**
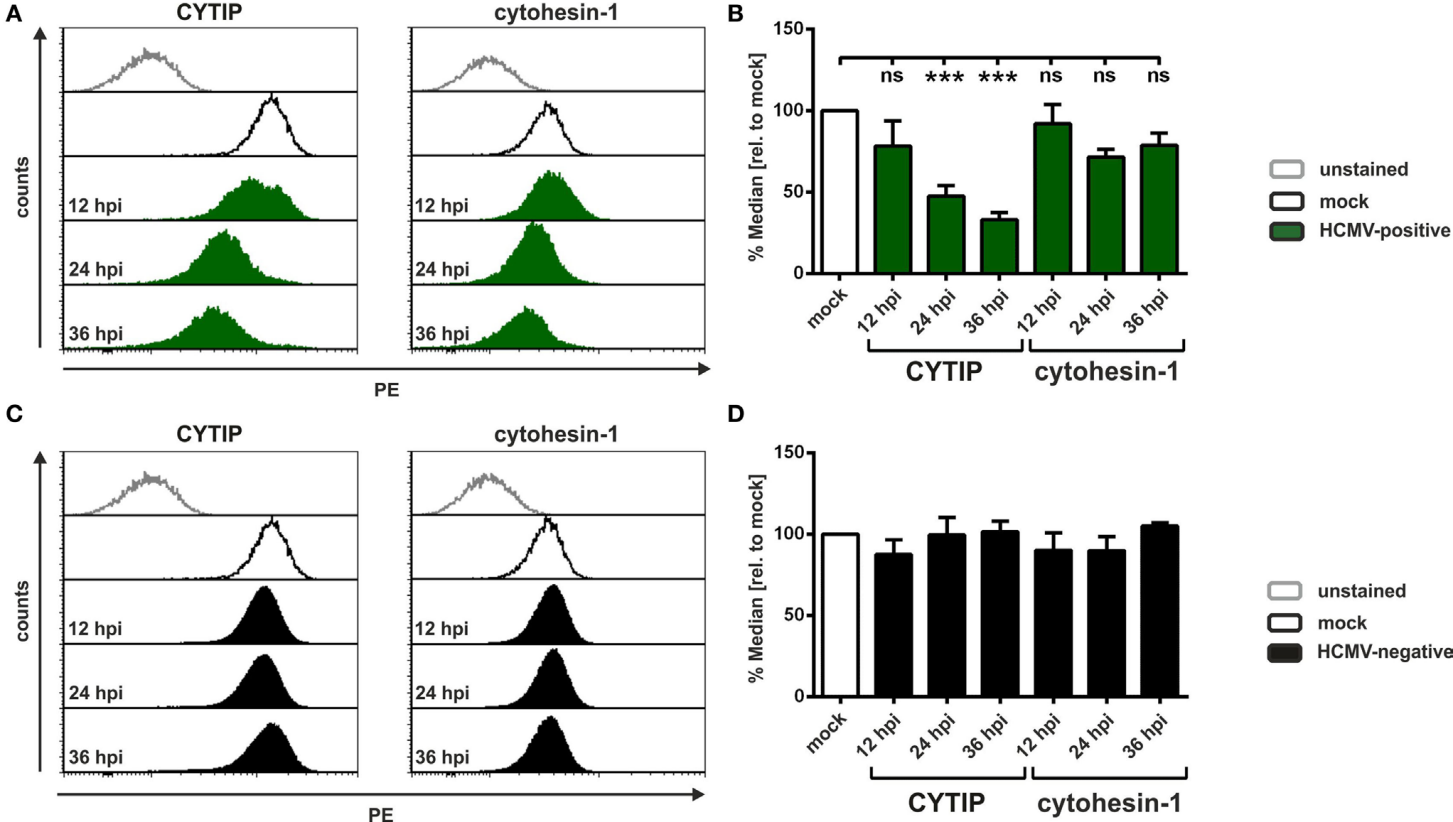
**Human cytomegalovirus (HCMV) induces CYTIP downmodulation early after infection**. Mature dendritic cells (mDCs) were mock- or HCMV-infected and harvested at the indicated time points post-infection. Cells were intracellularly stained with antibodies specific for CYTIP or cytohesin-1 and subjected to flow cytometric analyses. **(A,C)** Representative data of one donor are shown for CYTIP (left panel) or cytohesin-1 (right panel) expression levels 24 hpi. Mock conditions are depicted as black lines (exemplary for 24 hpi), **(A)** HCMV-positive cells as filled green histograms and **(C)** HCMV-negative cells as filled black histograms. **(B,D)** Quantification of CYTIP and cytohesin-1 protein levels 12 hpi (*n* = 3), 24 hpi (*n* = 6) and 36 hpi (*n* = 3). Median values of **(B)** HCMV-positive (green bars) and **(D)** HCMV-negative (black bars) mDCs were calculated relative to the respective mock condition (white bars). Significant changes (*** = *p* < 0.001) are marked by asterisks, non-significant changes (*p* > 0.05) as “ns.”

Since it is known that HCMV replicates slower than HSV-1, we analyzed CYTIP downregulation at different time points post-infection. Therefore, mDCs were mock- or HCMV-infected for the indicated time periods and subsequently subjected to intracellular flow cytometric analyses for detection of CYTIP and cytohesin-1 levels (Figure 5). One representative experiment for HCMV-positive (green histograms) and HCMV-negative (filled black histograms) mDCs is shown in Figures 5A,C, respectively. Summarized data out of at least three independent experiments are depicted as median values relative to mock in Figure 5B (HCMV-positive) and Figure 5D (HCMV-negative). CYTIP reduction is already observed 12 hpi in HCMV-positive mDCs, becoming statistically significant 24 hpi and even more prominent 36 hpi with a reduction of approximately 70% in comparison to mock-infected cells (Figure 5B). In contrast, cytohesin-1 is not downregulated to a significant extent, even 36 h post-HCMV infection. HCMV-negative mDCs showed no reduction in CYTIP and cytohesin-1 protein levels until 36 hpi (Figure 5D).

We, therefore, conclude that HCMV specifically downregulates CYTIP protein expression, while cytohesin-1 levels are not impaired.

### HCMV Induces a Proteasome-Dependent Degradation of CYTIP in mDCs

**Figure 6:**
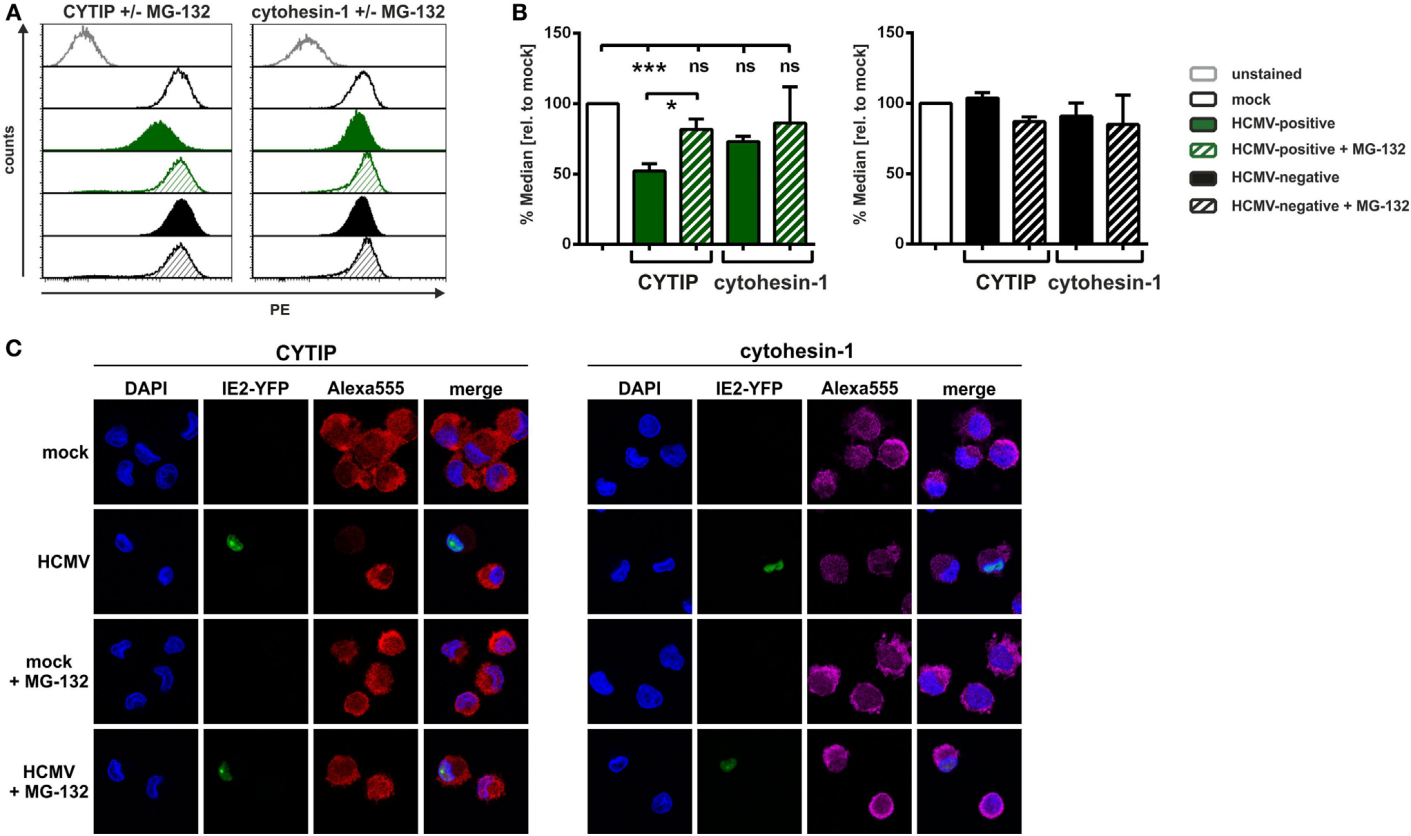
**In human cytomegalovirus (HCMV)-infected mature DCs (mDCs) CYTIP is degraded *via* the proteasome**. Mature dendritic cells were mock- or HCMV-infected, treated with or without MG-132 and harvested 24 hpi. **(A,B)** Subsequently, intracellular staining with antibodies specific for CYTIP or cytohesin-1 was performed, and samples were subjected to flow cytometric analyses. **(A)** One representative experiment is shown for CYTIP (left panel) or cytohesin-1 (right panel) levels. Mock conditions (black lined histograms), HCMV-positive cells (green filled histograms), and HCMV-negative cells (black filled histograms) are shown, while the respective MG-132-treated samples are depicted as striped histograms. **(B)** Summarized data of at least three independent experiments of CYTIP and cytohesin-1 protein levels, analyzed *via* intracellular flow cytometry. Median values of HCMV-positive mDCs with and without MG-132 treatment (green filled and green striped bars, respectively; left panel) as well as HCMV-negative mDCs with and without MG-132 treatment (black filled and black striped, respectively; right panel) were calculated relative to the respective mock conditions with and without MG-132 (white bars, set to 100%). Significant changes (*** = *p* < 0.001; * = *p* < 0.05) are marked by asterisks, non-significant changes (*p* > 0.05) as “ns.” **(C)** Immunofluorescence staining of CYTIP (left panel) and cytohesin-1 (right panel). The IE2-YFP fusion protein of the HCMV TB40E/IE2-EYFP strain allows direct determination of HCMV-positive and HCMV-negative cells. The nucleus was visualized using DAPI. The experiment was performed three times and representative data are shown.

We further investigated the mechanism by which HCMV mediates CYTIP downmodulation. Since our previous findings in HSV-1-infected mDCs showed a proteasome-dependent pathway of CYTIP degradation (45), we examined whether a similar mechanism is responsible for CYTIP downmodulation in the context of an HCMV infection. Thus, mDCs were mock- or HCMV-infected for 24 h, treated with or without the proteasome inhibitor MG-132 and analyzed for their CYTIP as well as cytohesin-1 expression *via* intracellular flow cytometry (Figures 6A,B). One representative experiment is depicted in Figure 6A, which shows CYTIP (left panel) and cytohesin-1 (right panel) protein levels in HCMV-positive (green histograms) and HCMV-negative (black histograms) mDCs 24 hpi. Figure 6B summarizes the flow cytometric data from at least three independent experiments.

Remarkably, CYTIP protein expression levels were restored in MG-132-treated HCMV-positive mDCs (green striped pattern) to approximately 80%. Regarding cytohesin-1, no significant differences in protein expression levels could be observed. CYTIP and cytohesin-1 protein levels are not affected in HCMV-negative mDCs compared to mock (Figure 6B, right panel). These observations are confirmed by immunofluorescence microscopy analyzing CYTIP and cytohesin-1 expression in mock- and HCMV-infected mDCs 24 hpi with or without MG–132 treatment (Figure 6C).

Taken together, our present study demonstrates that HCMV induces a proteasomal degradation of CYTIP in HCMV-positive mDCs early after infection. This leads to induced β2-integrin activation, and therefore increased mDC adhesion, which in turn inhibits CCL19-directed mDC transwell migration. Thus, not only the α-herpesvirus HSV-1 but also the β-herpesvirus HCMV uses this strategy to hamper the induction of antiviral immune responses. However, since HCMV does not downregulate the expression of CCR7, the reduction of CCL19-mediated migration is most likely due to induced adhesion.

## Discussion

Dendritic cells play a crucial role in the orchestration of immune responses by transferring the immunological status of peripheral tissues to areas with high lymphocyte density, i.e., to SLOs. There (naïve) T cells and/or B cells can be activated by presentation of antigens on DCs, in order to induce a potent immune response. Considering this, migration of DCs is a prerequisite for the successful initiation of adaptive immunity, important for elimination of e.g., viral infections. Particularly herpesviruses, which are able to establish lifelong persistent infections, have developed several strategies to counteract elimination by the immune system. In this study, we provide evidence that HCMV induces the activation of β2-integrins *via* the proteasomal degradation of the negative regulator CYTIP. This degradation of CYTIP is functionally relevant as we found induced adhesion and impaired transwell migration of HCMV-infected mDCs toward the chemokine CCL19, which is known to be predominantly expressed in lymph nodes. Therefore, immune evasion mechanisms targeting DC migration may allow HCMV to effectively evade cell-mediated immunity *in vivo*, supporting the production of new progeny virus and the establishment of latency.

Our finding that HCMV inhibits CCL19-mediated migration (Figure 1B) is the first report in which HCMV was described to reduce mDC migration toward this specific chemokine exclusively *via* the induction of adhesion, i.e., by increasing the activation status of β2-integrins (Figure 3). Thus, HCMV is able to not only inhibit upregulation of the respective chemokine receptor CCR7, when infecting iDCs prior to maturation (61), but also hamper migration toward CCL19 beyond CCR7-targeting, *via* the induction of adhesion upon HCMV infection of mDCs (Figures 3D,E). This very likely caused the reduced CCL19-mediated migration using fibronectin-coated membranes, as expression of the CCL19-receptor CCR7 was not affected by HCMV until 24 hpi (Figure 2B). Adhesion is mediated by integrins, thus we investigated the activation status of β2-integrins in uninfected or HCMV-infected mDCs, since our group previously showed that fibronectin adhesion of mDCs is predominantly mediated by the β2-integrin LFA-1 (45). Interestingly, HCMV-positive mDCs exhibit significantly increased activation of β2-integrins, similar to that of Mg/EGTA-treated mDCs, in comparison to mock cells (Figure 3C), which in turn resulted in a strong induction of fibronectin adhesion (Figure 3D). Additionally, adhesion of HCMV-infected mDCs on the LFA-1 specific ligand ICAM-1 was also strongly induced 24 hpi (Figure 3A). This hints toward a mechanism by which HCMV upregulates β2-integrin activation, i.e., LFA-1, and thus mDC adhesion in order to reduce migration. HSV-1-infected mDCs also showed impaired CCL19-mediated chemotaxis, however, to a far greater extent than for HCMV. This HSV-1-mediated inhibition of CCL19-specific-migration was not only due to stronger mDC adhesion upon HSV-1 infection, but also based on a decrease in CCR7 surface expression (44, 45).

In contrast to the reduced CCL19-mediated migration in HCMV-positive mDCs, we could not observe changes in CXCL12-mediated chemotaxis compared to mock, HCMV-negative or HCMV-UV-infected mDCs (Figure 1B). Strikingly, the surface expression of the respective chemokine receptor CXCR4 was found to be significantly induced on HCMV-positive mDCs (Figure 2D). Previous reports demonstrated that infection of different cell types with HCMV has diverse effects on CXCR4. Lecointe et al. showed that HCMV infection of microglial cells, but not of macrophages or astrocytes, leads to the downregulation of CXCR4 (62). Moreover, CXCL12-mediated migration of HCMV-infected cytotrophoblasts was found to be impaired, although CXCR4 surface expression was induced (63). Hence, it is tempting to speculate that HCMV may differentially regulate the CXCR4 axis in a cell-type specific manner. In addition, high expression of CXCL12, recognized by CXCR4, by osteoblasts in the bone marrow could explain why HCMV does not influence the migration toward this chemokine. In particular, HCMV is known to establish latency in CD34^+^ hematopoietic progenitor cells (39–41) and CD14^+^ monocytes (42, 43), and therefore, this directed migration of HCMV-infected mDCs to the bone marrow could stimulate dissemination and translocation to an ecological niche rich in potential target cells and thereby increase the reservoir of latently infected myeloid progenitor cells. Considering that HCMV uses multiple strategies to modulate DC migration by, e.g., regulating host chemokine receptor expression as well as expressing a plethora of chemokine and chemokine receptor homologs itself, it emphasizes the crucial role to attack the migratory ability of DCs to hamper early immune responses of the host.

The inside-out-signaling for β2-integrin activation is known to be based on intracellular binding of talin or cytohesin-1 to the CD18 subdomain and to membrane-bound PIP3 (27, 33, 64). Cytohesin-1 is a specific binding partner for the family of β2-integrins and highly expressed in mDCs (64). Consistent with our previous report on HSV-1-infected mDCs (45), cytohesin-1 protein expression was not significantly affected by HCMV infection (Figures 4 and 5). In contrast of directly regulating cytohesin-1 expression, the Kaposi’s sarcoma-associated herpesvirus cytohesin-1 homolog kaposin A was found to modulate the subcellular localization of cytohesin-1. Kaposin A directly interacts with cytohesin-1 to induce its membrane localization, finally leading to increased ICAM-1 adhesion (65). However, HCMV does not encode a kaposin A homolog, and thus, alternative mechanisms must be induced. Apart from a regulation of cytohesin-1 itself, we tested whether HCMV influences the expression of CYTIP, which is the direct interaction partner of cytohesin-1 and mediates the abrogation of cytohesin-1 induced LFA-1 activation (30). As recently shown by our group, the silencing of CYTIP indeed leads to induced mDC adhesion and reduced migration, hence CYTIP plays a crucial role in the regulation of adhesion and migration (45). Remarkably, we detected a strong proteasome-dependent degradation of CYTIP in HCMV-positive mDCs (Figure 6). Hence, we provide evidence that at least the prototypic members of α- and β-*herpesviridae*, HSV-1 and HCMV, respectively, share a similar mechanism to induce CYTIP degradation in order to induce adhesion and inhibit mDC migration.

The results presented here reveal that HCMV targets different aspects of DC biology, in particular adhesion and migration. By manipulating chemokine receptor expression and the activation status of integrins, HCMV impairs the migration of infected mDCs to block induction of antiviral immune responses. Further research concerning the molecular mechanism of CYTIP degradation in HCMV-infected mDCs and, thus, a better understanding of this viral immune evasive mechanism might lead to the development of new anti-herpesviral therapeutics.

## Ethics Statement

For the generation of monocyte-derived DCs from leukapheresis products of healthy donors, a positive vote from the local ethics committee has been obtained (reference number 4556). This study was carried out in accordance with the recommendations of the ethics committee of the “Friedrich-Alexander-Universität Erlangen-Nürnberg” with written informed consent from all subjects. All subjects gave written informed consent in accordance with the Declaration of Helsinki.

## Author Contributions

LG, AS, and CH designed the study and the experiments. LG, CD, PM, and LK performed experiments and analyzed and interpreted the data. VL and MT provided essential reagents. LG wrote the paper. CH, AS, PM, VL, and MT critically revised the manuscript. All authors approved the final version of the manuscript.

## Conflict of Interest Statement

The authors declare that the research was conducted in the absence of any commercial or finical relationships that could be construed as a potential conflict of interest.
